# Pseudoaneurysm associated haemosuccus pancreaticus - a rare and dangerous disease

**DOI:** 10.1186/s42155-020-00178-3

**Published:** 2020-11-19

**Authors:** Julia Wagenpfeil, Daniel Kütting, Christian P. Strassburg, Carsten Meyer

**Affiliations:** 1 Department of Radiology, University Hospital Bonn, Venusberg-Campus 1, 53127 Bonn, Germany; 2 Department of Gastroenterology, Hepatology, and Endocrinology, University Hospital Bonn, Venusberg-Campus 1, 53127 Bonn, Germany

**Keywords:** Haemosuccus pancreaticus, Upper gastrointestinal bleeding, Chronic pancreatitis, Coil and tissue adhesive (Histoacryl/ Lipiodol) embolization

## Abstract

**Background:**

In patients with upper gastrointestinal bleeding and hemorrhage originating from the major duodenal papilla pseudoaneurysm associated haemosuccus pancreaticus (HP) is a rare differential diagnosis which should be considered. Diagnosis may be challenging, as clinical presentation is often unspecific with only intermittent hemorrhage. Treatment of the causal pseudoaneurysm is mandatory and endovascular coil embolization is the suitable first-line management strategy.

Until now there are only a very few studies about this clinical picture and its therapeutic options, especially data regarding whether additional fluid embolization is beneficial/necessary in HP is currently lacking.

**Case presentation:**

We report a case of a 59-year-old male patient with chronic pancreatitis and haemosuccus pancreaticus caused by a pancreatico-arterial fistula with an associated inflammatory pseudoaneurysm of the splenic artery. Initially we sought to embolize the pseudoaneurysm with microcoils. As only one coil could be safely deployed in the pseudoaneurysm we additionally employed tissue adhesive embolization in order to achieve complete occlusion of the pseudoaneurysm as well as the pancretico-arterial fistula. In the presented case inflammatory levels decreased following embolization, possibly linked to a decline in pathologic excretion of elastase and autodigestion. As not only the pseudoaneurysm but also the underlying fistula were occluded, the risk of recurrence may conceivably be reduced.

**Conclusions:**

Diagnosis of HP is difficult and treatment of the causal pseudoaneurysm is mandatory. Endovascular embolization is the suitable first-line management strategy, complete occlusion of the fistula should be considered when possible.

## Introduction

Haemosuccus pancreaticus (HP), a rare cause of upper gastrointestinal bleeding (1:1500 cases of gastrointestinal hemorrhage) (Subasinghe et al. 2012), is defined as hemorrhage originating from the pancreas, pancreatic duct, or (less frequently) from peri-pancreatic vessels (e. g. splenic artery) into the pancreatic duct. This disease is typically found in patients with chronic pancreatitis (Vimalraj et al. 2009). Increased excretion of elastase, induced by chronic local inflammation, leads to autodigestion of peripancreatic vessels or erosion of concomitant pseudocysts into adjacent vessels (Stanley et al. 1976). Diagnosis is challenging and frequently delayed, as hemorrhage is usually only intermittent and not severe enough to cause hemodynamic instability (Mandaliya et al. 2014). Clinical symptoms are similar to those typically found in other forms of gastrointestinal bleeding and include non-specific epigastric pain, hematemesis, melena and hyperamylasemia. Haemosuccus pancreaticus is associated with a high mortality rate, therapeutic options consist of selective radiological embolization or surgery (Subasinghe et al. 2012).

## Case report

A 59-year-old male patient with a history of alcohol induced recurrent pancreatitis and infected pseudocysts initially presented to an academic teaching hospital with fatigue, exertional dyspnea and reduced capacity. Laboratory analysis demonstrated hypochromic microcytic anemia (hemoglobin: 6.3 g/dl), initial computed tomography of the abdomen was deemed unremarkable. Esophagogastroduodenoscopy detected an active hemorrhage from the major duodenal papilla, which could not be reproduced in the following angiography. As the cause of haemosuccus pancreaticus remained unclear, the patient was transferred to our maximum care hospital for further diagnosis and treatment of the upper gastrointestinal bleeding.

**Fig. 1:**
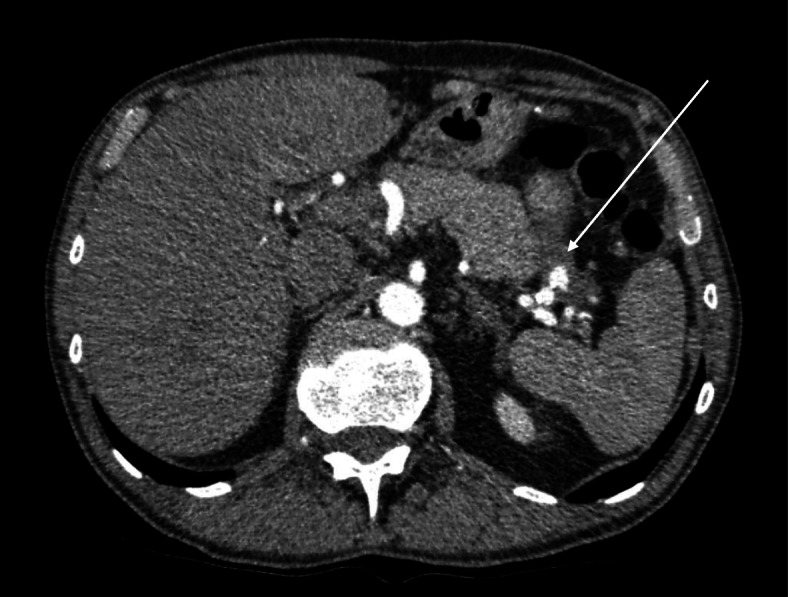
Pseudoaneurysm of the splenic artery

Repeat esophagogastroduodenoscopy showed gastric varices but was otherwise unremarkable. During re-evaluation of the initial computed tomography a pseudoaneurysm of the splenic artery was discovered, sizing 7 × 8 × 9 mm (Fig. 1).

**Fig. 2:**
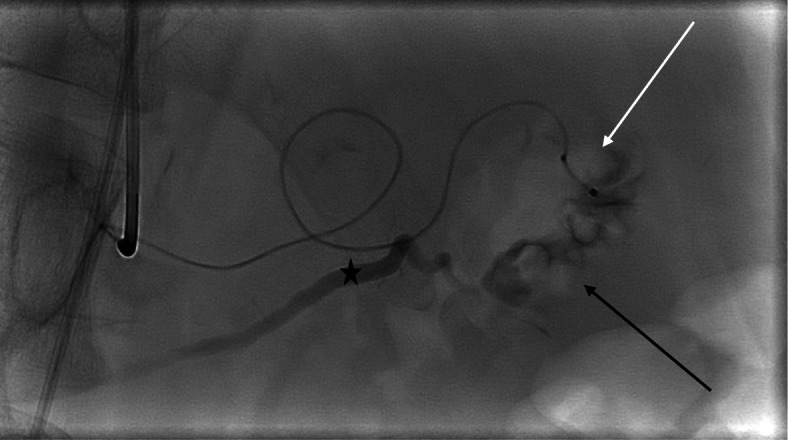
Microcatheter placed in pseudoaneurysm (white arrow), pancreatico-arterial fistula (black arrow), opacified pancreatic duct (asterisks)

Angiography was performed again to verify and potentially embolize the pseudoaneurysm. The splenic artery was catheterized selectively with a 4 Fr catheter via right femoral access. Initial angiogram demonstrated spontaneous occlusion of the pseudoaneurysm. As both clinical and laboratory signs of hemorrhage had persisted the days prior to intervention, decision was made to super-selectively catheterize and potentially embolize the presumably occluded aneurysm. Angiogram following successful catheterization revealed a fistula between the pseudoaneurysm and the main pancreatic duct (Fig. 2).

**Fig. 3:**
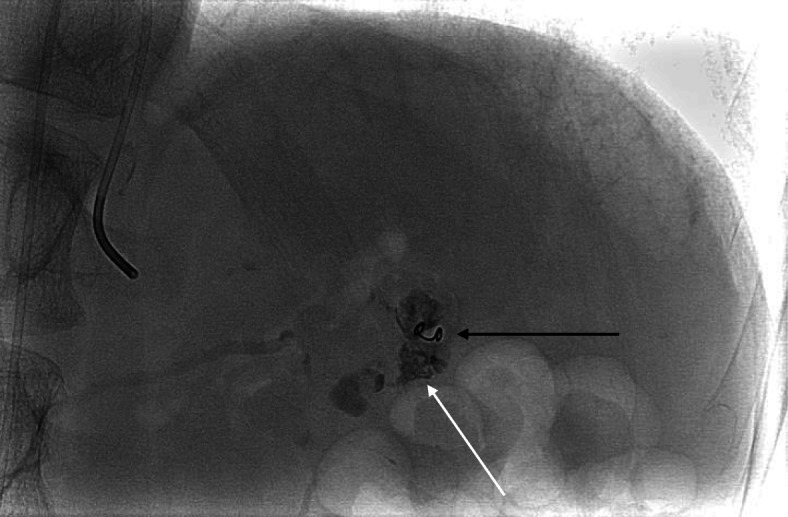
Coil embolization by 2 × 20 mm HILAL Microcoil (black arrow) and proper distribution of tissue adhesive (white arrow)

Due to pre-existing spontaneous partial thrombosis only one microcoil (HILAL 2 × 20 mm, Cook, Bjaeverskov, Denmark) could be safely placed within the aneurysm. In order to completely occlude the pancreatico-arterial fistula additional tissue adhesive embolization was performed by Histoacryl (B. Braun, Melsungen, Germany)/Lipiodol (Guerbet, Aulnay-sous-Bois, France); ratio 1:5; 0,4 ml). Distribution of tissue adhesive in the pseudoaneurysm and fistula is demonstrated in Fig. 3.

Final angiogram confirmed complete obstruction of the pseudoaneurysm, as well as preserved regular perfusion of the splenic artery. Subsequent follow-up computed tomography performed 4 days and 10 months after embolization demonstrated hyperdense tissue adhesive in the fistula/aneurysmal sac as well as persistent occlusion of the pseudoaneurysm. The postinterventional course was uneventful and the patient was discharged in the following days. He was re-admitted 6 months after initial intervention: Endoscopy revealed bleeding from gastric varices, the papilla appeared unremarkable.

## Discussion

Haemosuccus pancreaticus is a rare complication of acute or chronic pancreatitis, predominantly observed in men (sex ratio 7:1), associated with chronic alcohol consumption (Vimalraj et al. 2009). Clinical symptoms include upper gastrointestinal bleeding, non-specific epigastric pain, hematemesis and melena (Singh et al. 2016). As hemorrhage is usually only intermittent and not severe enough to cause hemodynamic instability, diagnosis is frequently delayed and difficult to make (Vimalraj et al. 2009), as observed in our reported case.

Although endoscopy may reveal bleeding from the papilla, the actual source will typically remain unclear. Nevertheless, other significant causes of upper gastrointestinal bleeding (e.g. erosive gastritis, esophageal and gastric fundus varices or ulcers) may be excluded. Contrast enhanced computed tomography allows for reliable detection of pancreatic pathologies, as well as the assessment of potential complications of chronic pancreatitis while visualizing the peripancreatic vessels. Finally, angiographic intervention (including coil/glue embolization) is the therapy of choice for gastrointestinal bleeds not manageable by endoscopy (Yoshida et al. 2018). Haemosuccus pancreaticus, especially if caused by a pseudoaneurysm, is a potentially life-threatening disease (Subasinghe et al. 2012). Both interventional radiological and surgical approaches are proven therapeutic options. Parent vessel occlusion by means of coil embolization is the most frequently described technique to treat pseudoaneurysm associated HP (Yoshida et al. 2018). This technique was avoided in the current case due to the risk of splenic infarction. Covered stents, an alternative option in the management of pseudoaneurysms arising from larger vessels, offer the advantage of preserving arterial flow and can be used in selective cases to bridge time until elective surgery (De Rosa et al. 2012). However, active pancreatico-arterial fistulas, as suspected in the current case, may lead to graft infections. Furthermore, stent/stent graft delivery into peripheral pseudoaneurysms is technically challenging due to the typically torturous course of the splenic artery. In this regard, Benz et al. described the first successful implantation of an uncoated metal palmaz stent across the aneurysmal segment of the splenic artery for treatment of pseudoaneurysm (Benz et al. 2000).

The probability of a recurrent bleeding after catheter assisted embolization varies between 0 and 30% depending on the reference literature (Vimalraj et al. 2009). In order to completely occlude the pancreatico-arterial fistula we additionally employed tissue adhesive embolization (Histoacryl/Lipiodol; ratio 1:5; 0,4 ml). By doing so, we did not only occlude the pseudoaneurysm, but also the underlying fistula. Ideally, this also stopped pathologic excretion of elastase and autodigestion, thus possibly reducing the risk of recurrence. The patient additionally received trans-splenic portal venous reconstruction and trans jugular porto-systemic shunt 16 months following embolization of splenic artery pseudoaneurysm. In follow up imaging the pseudoaneurysm remained occluded, no further episodes of HP were reported. Data regarding additional fluid embolization in such cases is sparse. Although the current results are promising, the risk of splenic infarction and dislocation or superinfection of glue has to keep in mind.

In patients with persistent unstable hemodynamics, recurrent bleeding or failed embolization, surgical management should be instituted without delay (Vimalraj et al. 2009).

## Conclusion

In patients with upper gastrointestinal bleeding and hemorrhage originating from the major duodenal papilla pseudoaneurysm associated HP is a rare differential diagnosis which should be considered. These patients are difficult to manage, frequently present with recurrent bleeding and have a high mortality rate. Treatment of the causal pseudoaneurysm is mandatory and endovascular embolization is the suitable first-line management strategy, complete occlusion of the fistula should be considered when possible.

## Data Availability

The datasets generated and/or analysed during the current study are available from the corresponding author on reasonable request.
